# Supplementary material for: Practices of Rapid Sequence Induction for Prevention of Aspiration—An International Declarative Survey
Source: J Clin Med. 2025 Mar 22;14(7):2177. doi: 10.3390/jcm14072177 (PMC11989417; doi:10.3390/jcm14072177)
Supplement: Supplementary file 1 [file jcm-14-02177-s001.zip › jcm-3379469-Supplementary File S1.pdf]

Q1 How do you define a Rapide Sequence Induction during anesthesia at risk for aspiration? (several possible answers)

Réponses obtenues : 494 Question(s) ignorée(s) : 0

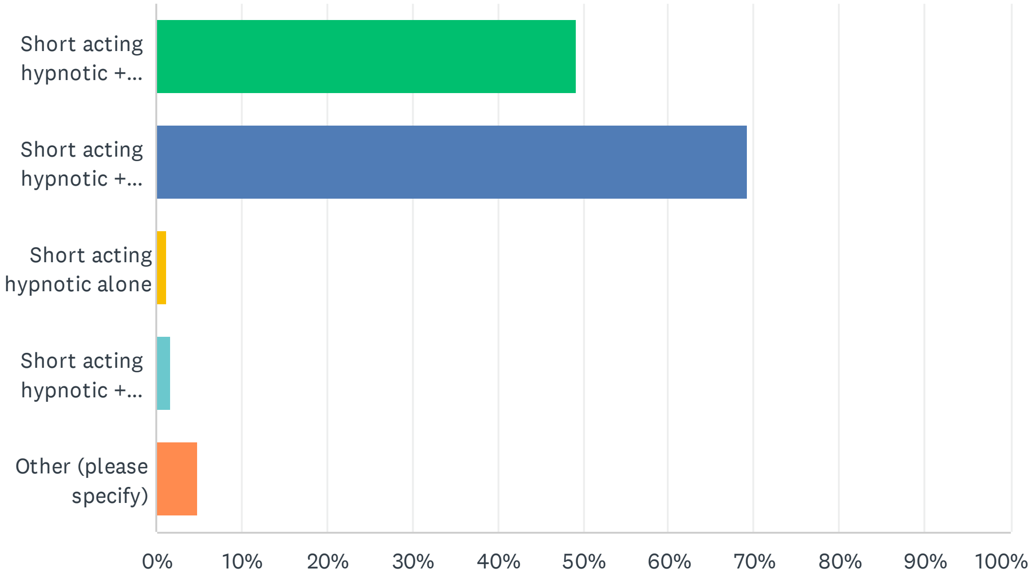

CHOIX DE RÉPONSES	RÉPONSES	
Short acting hypnotic + Short acting neuromuscular blocker (succynilcholine or rocuronium)	49.19%	243
Short acting hypnotic + short acting neuromuscular blocker + opioid	69.23%	342
Short acting hypnotic alone	1.21%	6
Short acting hypnotic + opioid	1.62%	8
Other (please specify)	4.86%	24
Nombre total de participants: 494		

Q2 If you use opioids for rapide sequence induction, in which cases?

Réponses obtenues : 421 Question(s) ignorée(s) : 73

Q3 How old are you (years)?

Réponses obtenues : 460 Question(s) ignorée(s) : 34

Q4 What is your country?

Réponses obtenues : 461 Question(s) ignorée(s) : 33

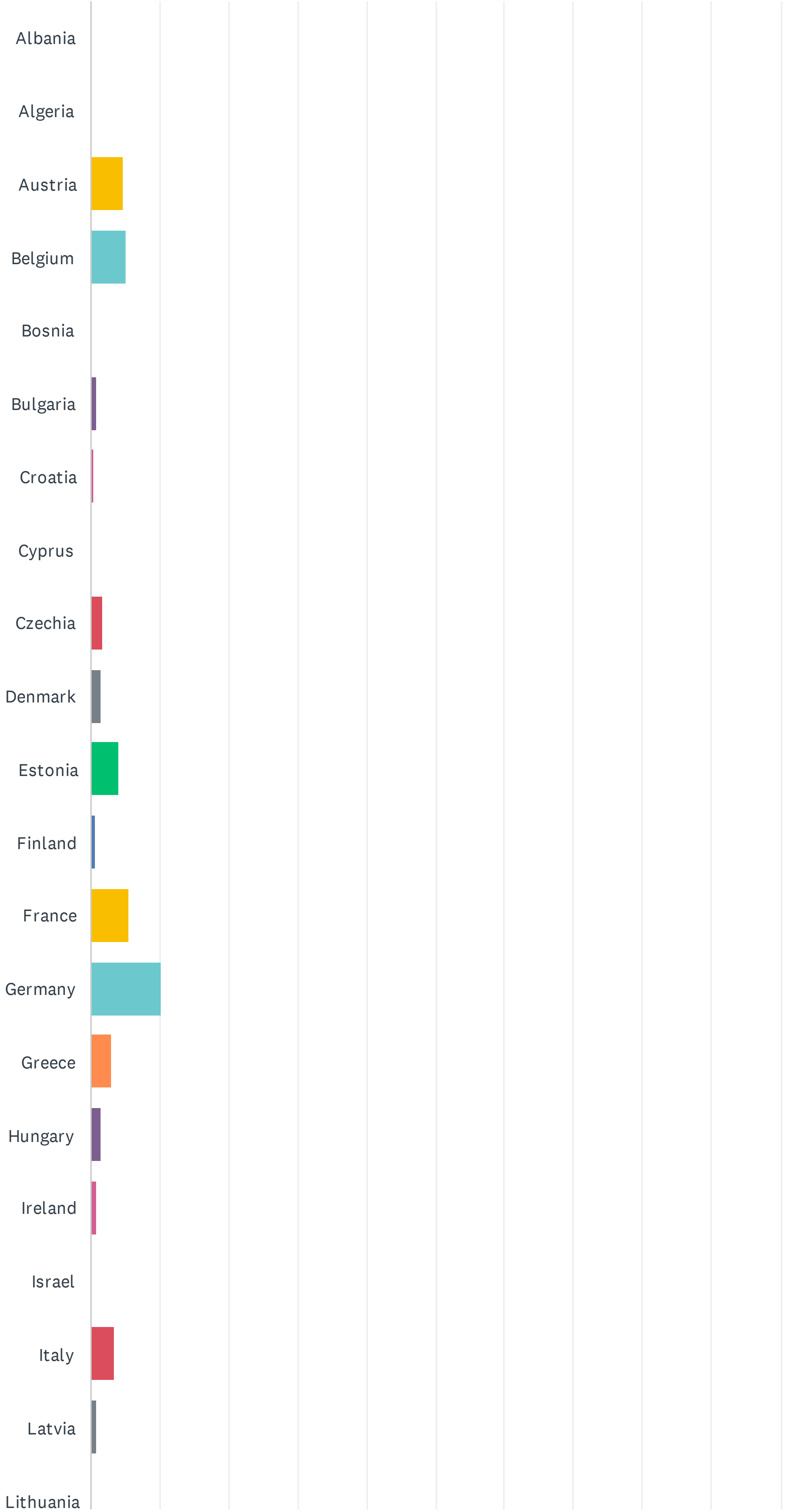
Albania
Algeria
Austria
Belgium
Bosnia
Bulgaria
Croatia
Cyprus
Czechia
Denmark
Estonia
Finland
France
Germany
Greece
Hungary
Ireland
Israel
Italy
Latvia
Lithuania

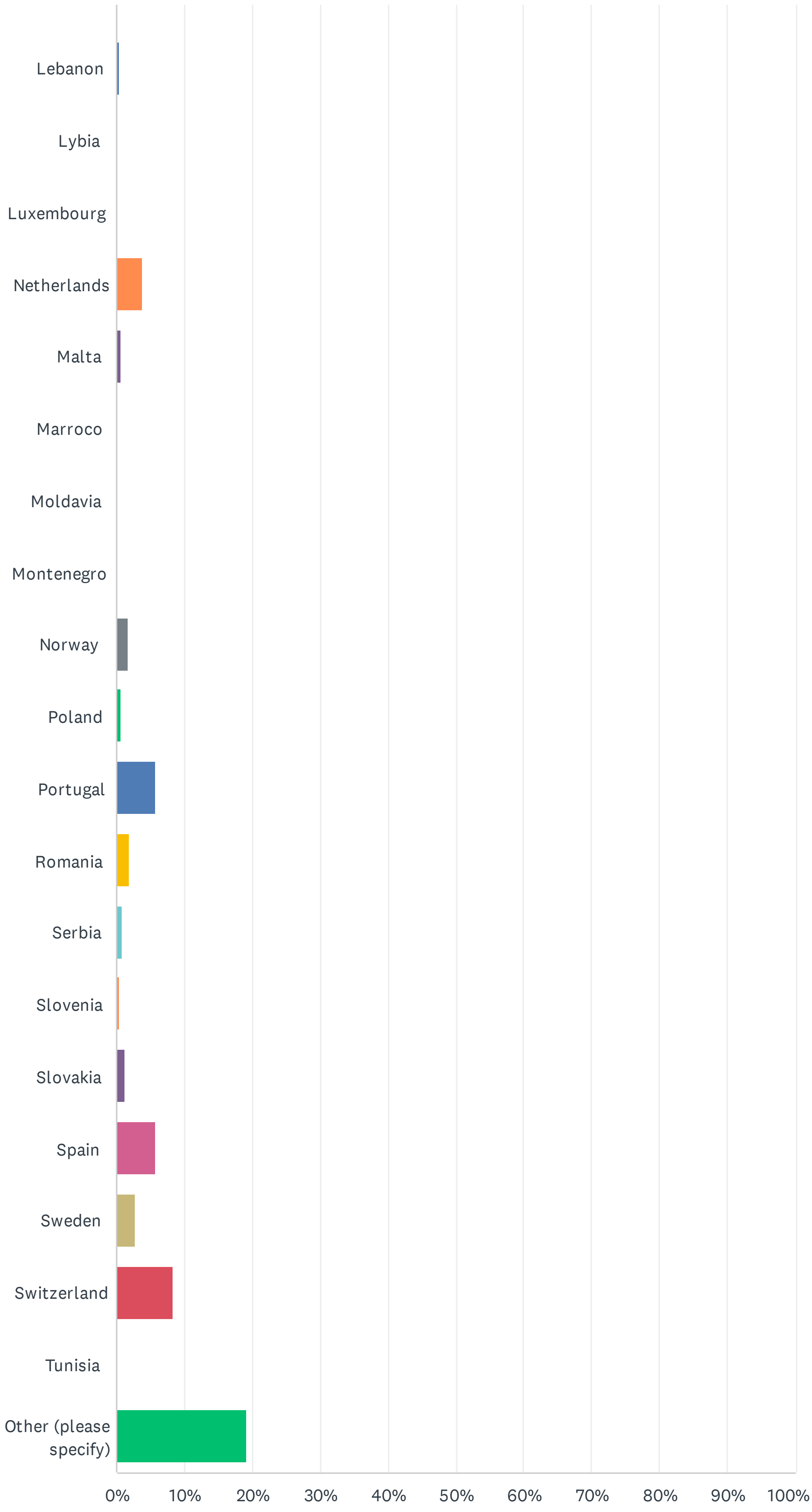
Lebanon
Lybia
Luxembourg
Netherlands
Malta
Marroco
Moldavia
Montenegro
Norway
Poland
Portugal
Romania
Serbia
Slovenia
Slovakia
Spain
Sweden
Switzerland
Tunisia
Other (please specify)
0%
10%
20%
30%
40%
50%
60%
70%
80%
90%
100%

CHOIX DE RÉPONSES	RÉPONSES	
Albania	0.00%	0
Algeria	0.00%	0
Austria	4.77%	22
Belgium	5.21%	24
Bosnia	0.22%	1
Bulgaria	0.87%	4
Croatia	0.43%	2
Cyprus	0.22%	1
Czechia	1.74%	8
Denmark	1.52%	7
Estonia	4.12%	19
Finland	0.65%	3
France	5.64%	26
Germany	10.20%	47
Greece	3.04%	14
Hungary	1.52%	7
Ireland	0.87%	4
Israel	0.22%	1
Italy	3.47%	16
Latvia	0.87%	4
Lithuania	0.22%	1
Lebanon	0.43%	2
Lybia	0.22%	1
Luxembourg	0.22%	1
Netherlands	3.90%	18
Malta	0.65%	3
Marroco	0.00%	0
Moldavia	0.00%	0
Montenegro	0.00%	0
Norway	1.74%	8
Poland	0.65%	3
Portugal	5.86%	27

Romania	1.95%	9
Serbia	0.87%	4
Slovenia	0.43%	2
Slovakia	1.30%	6
Spain	5.86%	27
Sweden	2.82%	13
Switzerland	8.24%	38
Tunisia	0.00%	0
Other (please specify)	19.09%	88
TOTAL		461

Q5 What is your professional position?

Réponses obtenues : 462 Question(s) ignorée(s) : 32

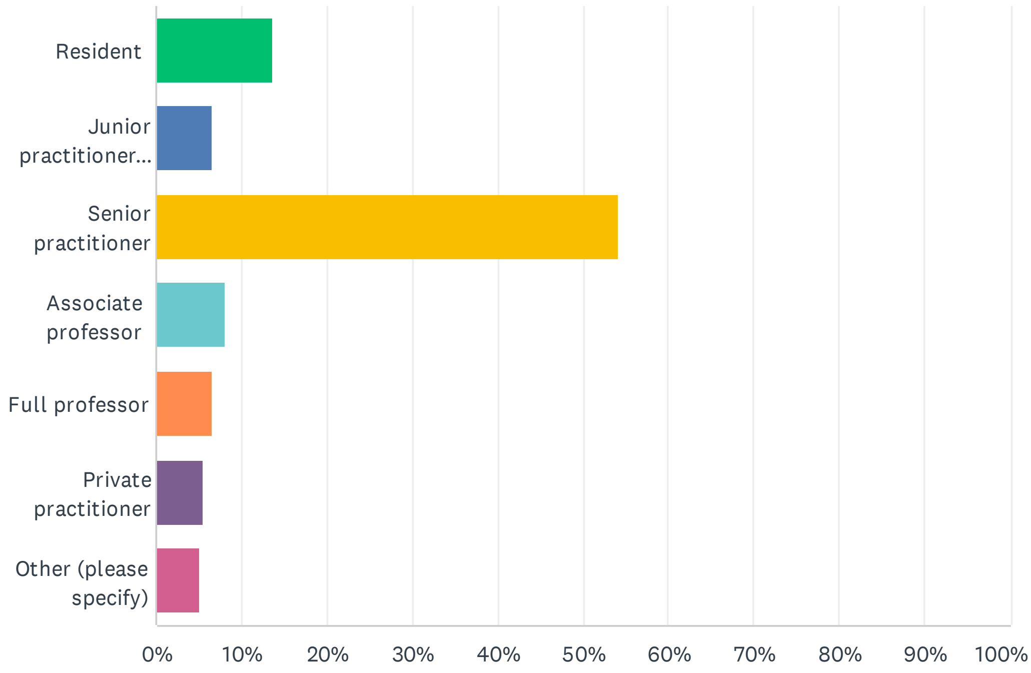

CHOIX DE RÉPONSES	RÉPONSES	
Resident	13.64%	63
Junior practitioner (2 years after residency)	6.71%	31
Senior practitioner	54.11%	250
Associate professor	8.01%	37
Full professor	6.71%	31
Private practitioner	5.63%	26
Other (please specify)	5.19%	24
TOTAL		462

Q6 How many years of professional experience in anesthesia/ICU/Emergency (including residency)?

Réponses obtenues : 463 Question(s) ignorée(s) : 31

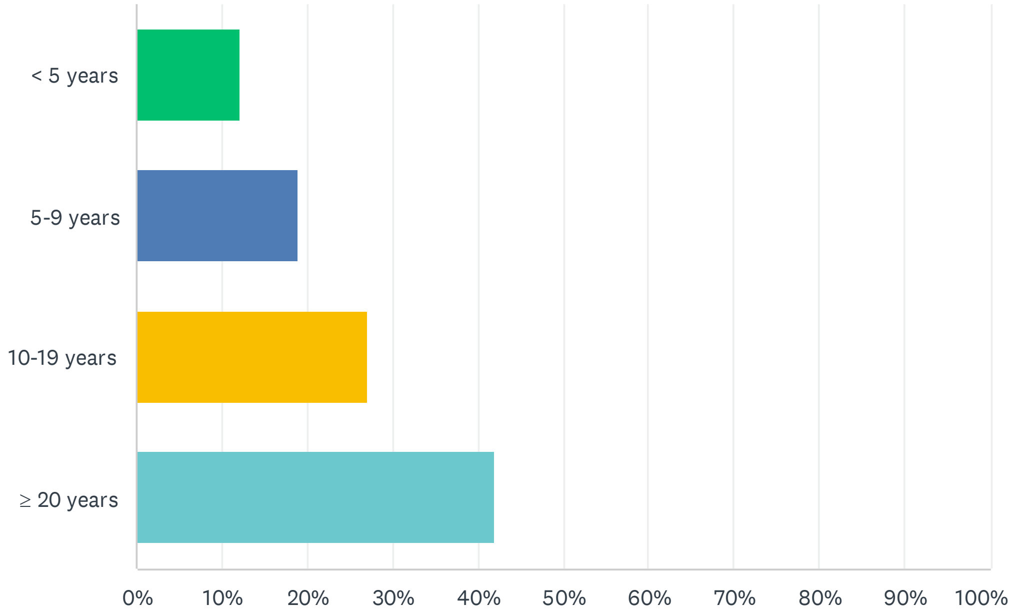

CHOIX DE RÉPONSES	RÉPONSES	
< 5 years	12.10%	56
5-9 years	19.01%	88
10-19 years	27.00%	125
≥ 20 years	41.90%	194
TOTAL		463

Q7 What is your usual facility?

Réponses obtenues : 463 Question(s) ignorée(s) : 31

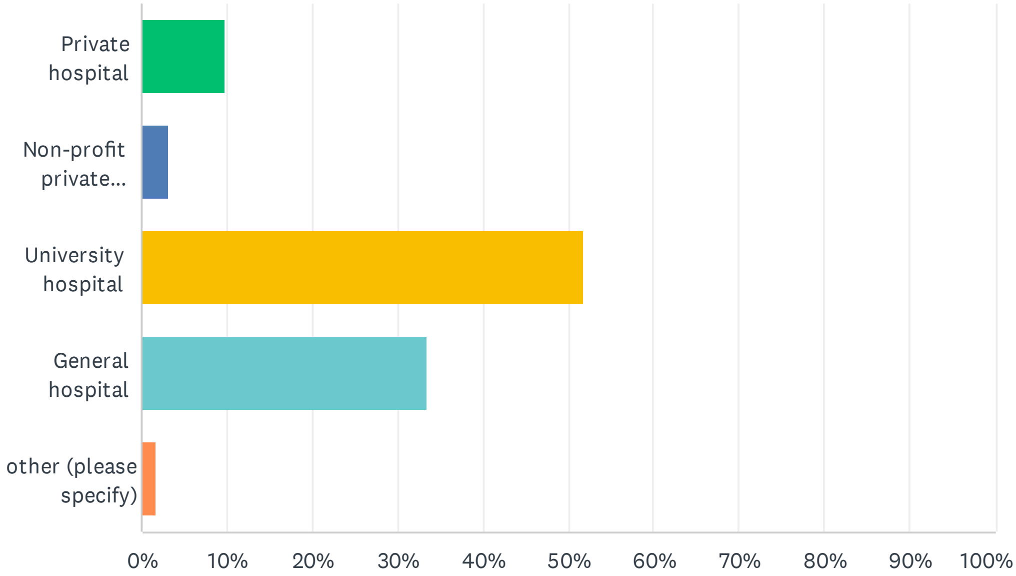

CHOIX DE RÉPONSES	RÉPONSES	
Private hospital	9.72%	45
Non-profit private hospital	3.24%	15
University hospital	51.84%	240
General hospital	33.48%	155
other (please specify)	1.73%	8
TOTAL		463

Q8 What is your main professional activity (only one possible answer)

Réponses obtenues : 463 Question(s) ignorée(s) : 31

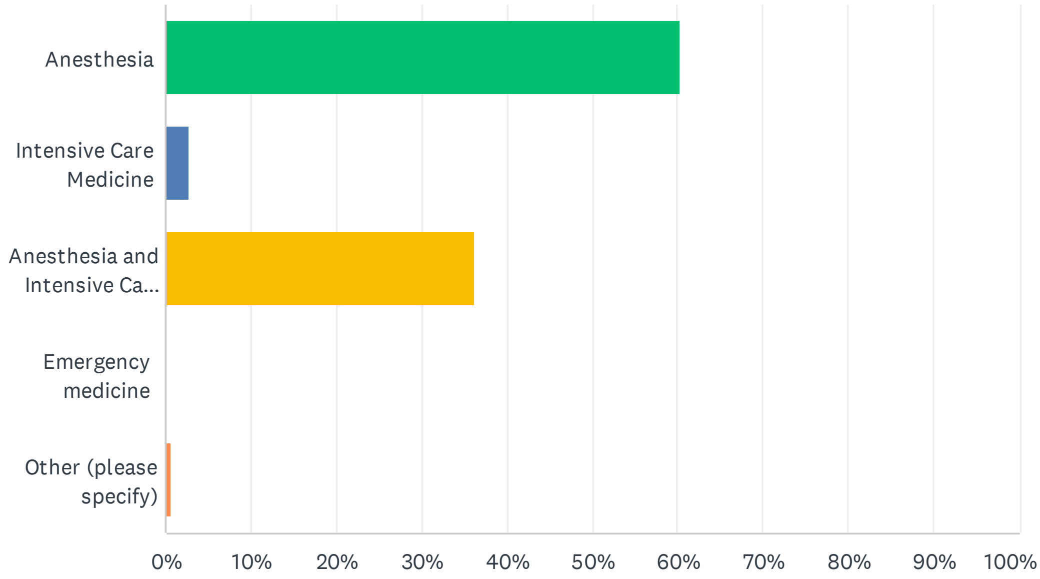

CHOIX DE RÉPONSES	RÉPONSES	
Anesthesia	60.26%	279
Intensive Care Medicine	2.81%	13
Anesthesia and Intensive Care Medicine (mixed activity)	36.29%	168
Emergency medicine	0.00%	0
Other (please specify)	0.65%	3
TOTAL		463

Q9 How many anesthetic induction(s) do you perform per week?

Réponses obtenues : 462 Question(s) ignorée(s) : 32

Q10 For what type of procedure? (several possible answers)

Réponses obtenues : 463 Question(s) ignorée(s) : 31

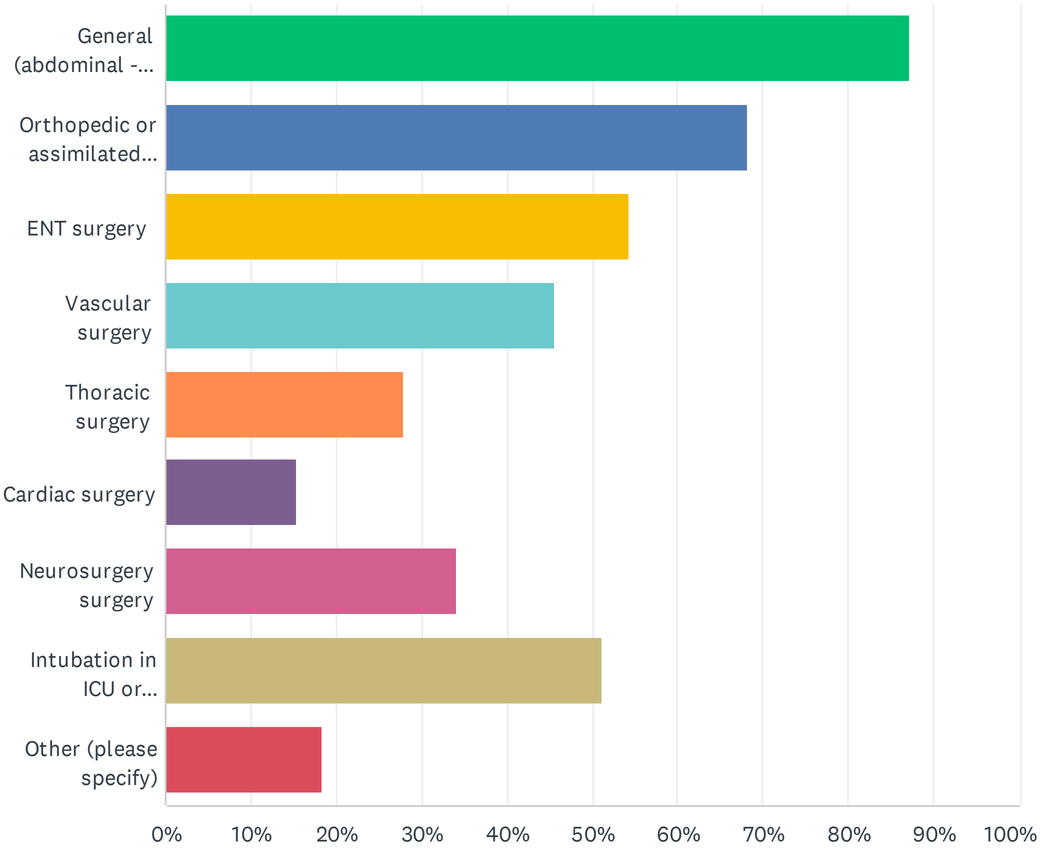

CHOIX DE RÉPONSES	RÉPONSES	
General (abdominal - urology - gynecologic) surgery	87.26%	404
Orthopedic or assimilated surgery	68.25%	316
ENT surgery	54.43%	252
Vascular surgery	45.57%	211
Thoracic surgery	27.86%	129
Cardiac surgery	15.33%	71
Neurosurgery surgery	34.13%	158
Intubation in ICU or Emergency Departement	51.19%	237
Other (please specify)	18.36%	85
Nombre total de participants: 463		

Q11 Do you manage anesthesia for urgent abdominal surgery?

Réponses obtenues : 463 Question(s) ignorée(s) : 31

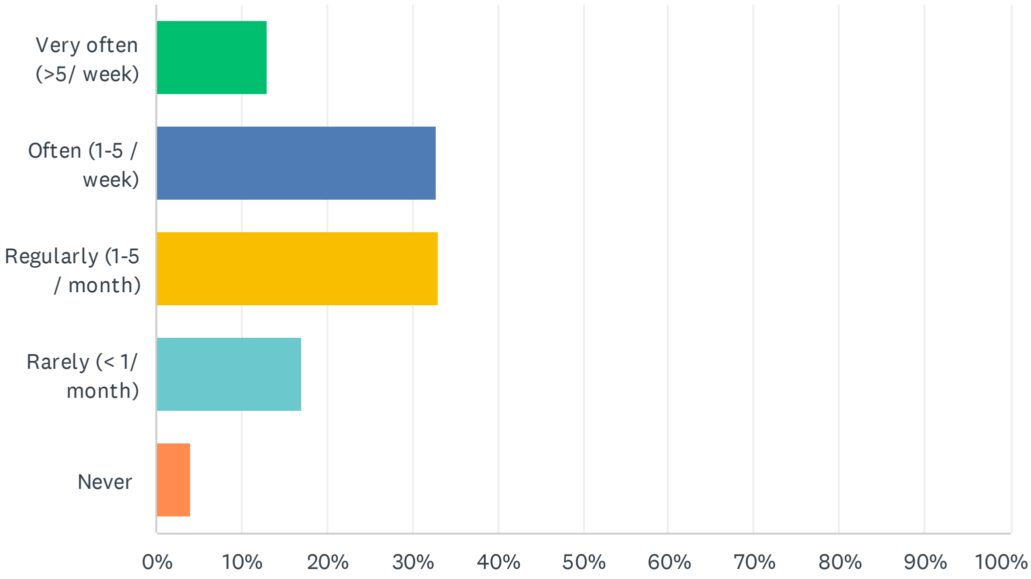

CHOIX DE RÉPONSES	RÉPONSES	
Very often (>5/ week)	12.96%	60
Often (1-5 / week)	32.83%	152
Regularly (1-5 / month)	33.05%	153
Rarely (< 1/ month)	17.06%	79
Never	4.10%	19
TOTAL		463

Q12 Do you manage anesthesia for trauma?

Réponses obtenues : 463 Question(s) ignorée(s) : 31

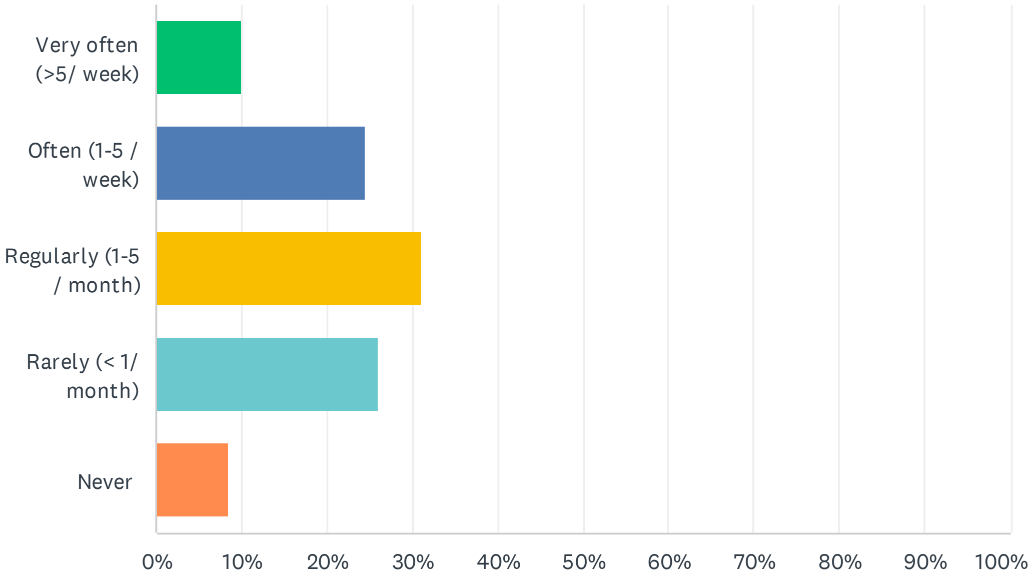

CHOIX DE RÉPONSES	RÉPONSES	
Very often (>5/ week)	9.94%	46
Often (1-5 / week)	24.62%	114
Regularly (1-5 / month)	31.10%	144
Rarely (< 1/ month)	25.92%	120
Never	8.42%	39
TOTAL		463

Q13 Do you manage anesthesia for obstetric?

Réponses obtenues : 462 Question(s) ignorée(s) : 32

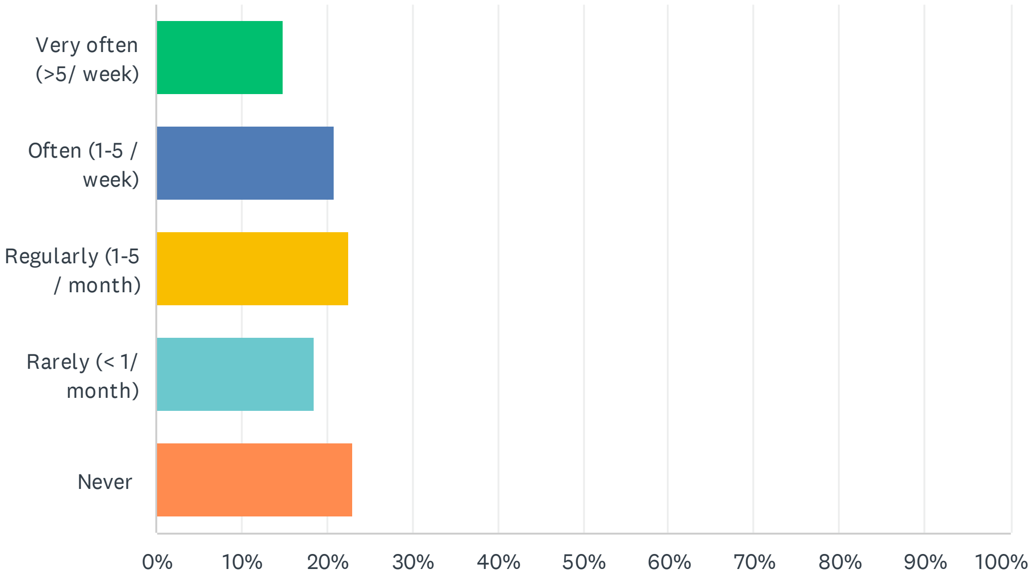

CHOIX DE RÉPONSES	RÉPONSES	
Very often (>5/ week)	14.94%	69
Often (1-5 / week)	21.00%	97
Regularly (1-5 / month)	22.51%	104
Rarely (< 1/ month)	18.61%	86
Never	22.94%	106
TOTAL		462

Q14 When performing a rapid sequence induction, do you use? (several possible answers)

Réponses obtenues : 459 Question(s) ignorée(s) : 35

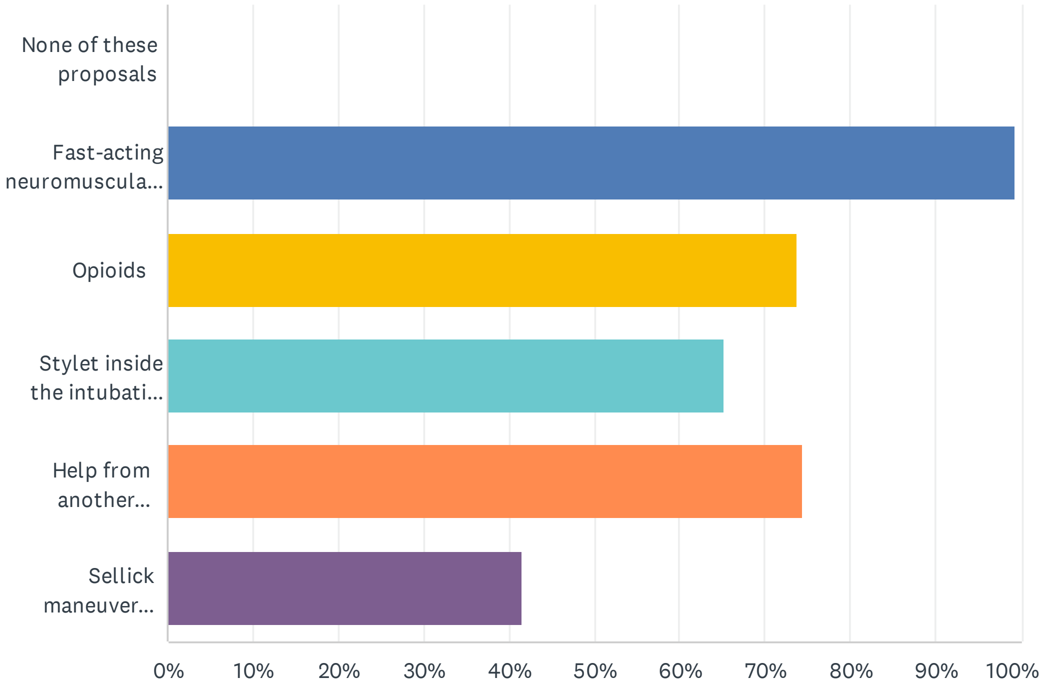

CHOIX DE RÉPONSES	RÉPONSES	
None of these proposals	0.00%	0
Fast-acting neuromuscular blockade (succinylcholine or rocuronium at high doses)	99.35%	456
Opioids	73.86%	339
Stylet inside the intubation probe	65.14%	299
Help from another anesthesia provider (physician, resident or anesthesia nurse)	74.51%	342
Sellick maneuver (cricoid pressure)	41.61%	191
Nombre total de participants: 459		

Q15 Concerning preoxygenation before rapid sequence induction, do you use? (several possible answers)

Réponses obtenues : 458 Question(s) ignorée(s) : 36

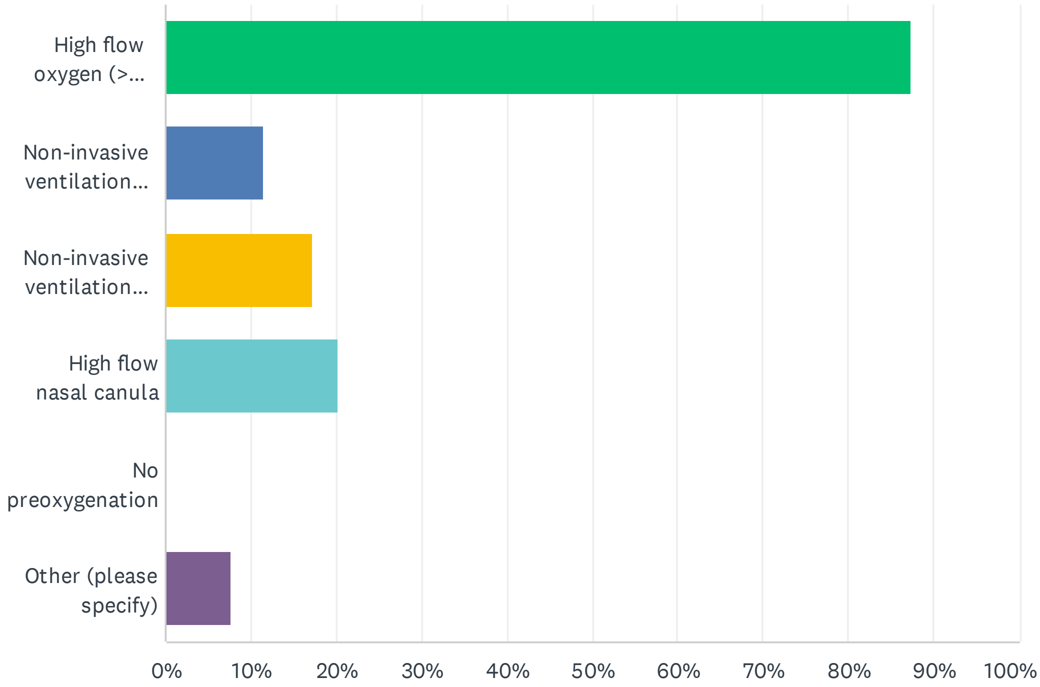

CHOIX DE RÉPONSES	RÉPONSES	
High flow oxygen (> 10L/min), no PEEP, no pressure support	87.34%	400
Non-invasive ventilation with pressure support, no PEEP (< 3 cmH2O)	11.57%	53
Non-invasive ventilation with pressure support AND PEEP (>= 3 cmH2O)	17.25%	79
High flow nasal canula	20.31%	93
No preoxygenation	0.00%	0
Other (please specify)	7.64%	35
Nombre total de participants: 458		

Q16 Do you perform gastric echography in the decision process to perform or not a rapid sequence induction?

Réponses obtenues : 459   Question(s) ignorée(s) : 35

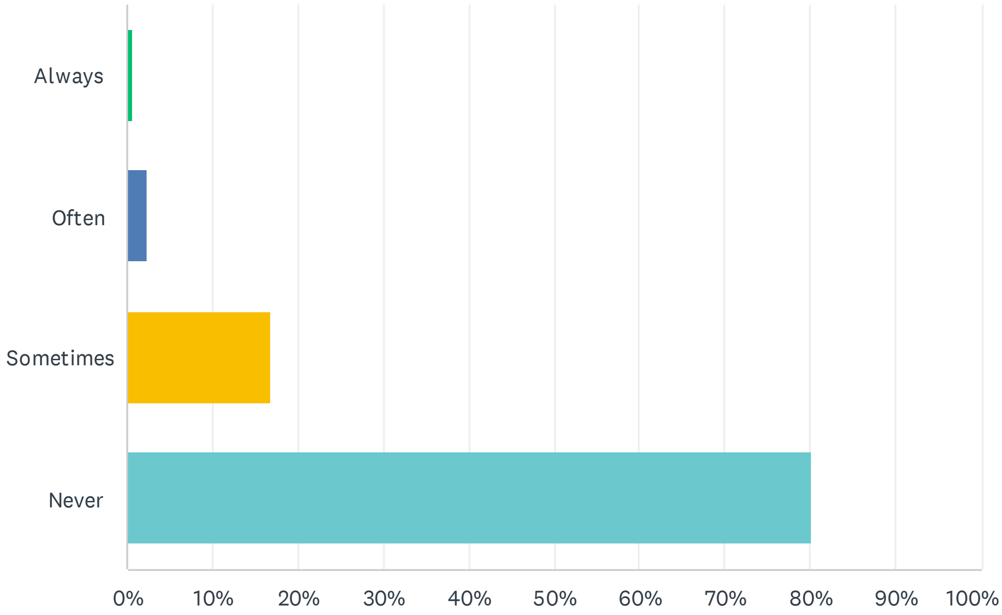

CHOIX DE RÉPONSES	RÉPONSES	
Always	0.65%	3
Often	2.40%	11
Sometimes	16.78%	77
Never	80.17%	368
TOTAL		459

Q17 If you never use gastric echography, why?

Réponses obtenues : 320   Question(s) ignorée(s) : 174

Q18 You take charge of a 43 year old patient for a simple appendectomy, operated on by laparoscopy. Fasting since the day before, this patient has never vomited. How do you manage this case?

Réponses obtenues : 447   Question(s) ignorée(s) : 47

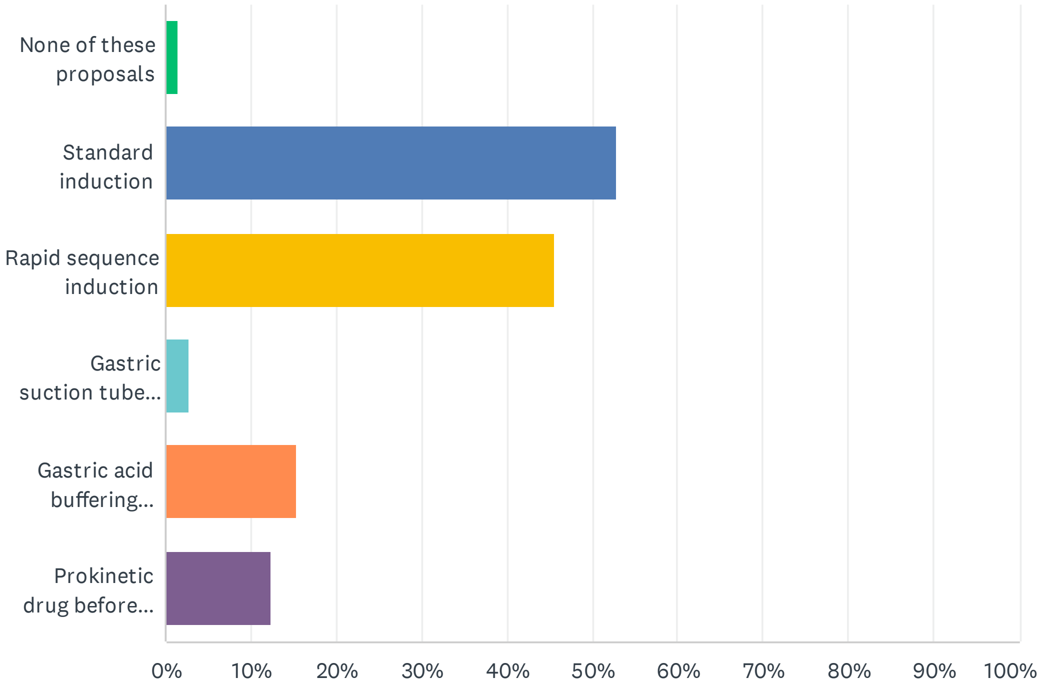

CHOIX DE RÉPONSES	RÉPONSES	
None of these proposals	1.57%	7
Standard induction	52.80%	236
Rapid sequence induction	45.64%	204
Gastric suction tube before anesthesia	2.68%	12
Gastric acid buffering before anesthesia (for example ranitidine/cimetidine)	15.44%	69
Prokinetic drug before anesthesia (for example erythromycin or metoclopramide)	12.30%	55
Nombre total de participants: 447		

Q19 You take charge of a 22 year old patient with a bi-malleolar fracture at 9:30 am (last solid meal at 7:30 am the same day). How do you manage this case?

Réponses obtenues : 447 Question(s) ignorée(s) : 47

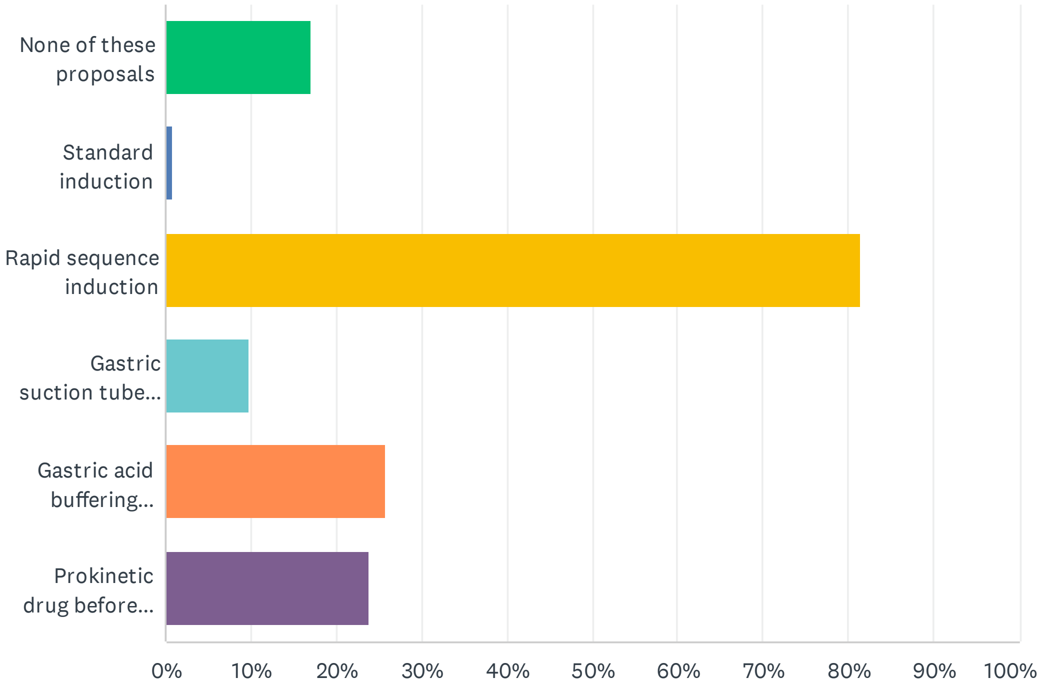

CHOIX DE RÉPONSES	RÉPONSES	
None of these proposals	17.00%	76
Standard induction	0.89%	4
Rapid sequence induction	81.43%	364
Gastric suction tube before anesthesia	9.84%	44
Gastric acid buffering before anesthesia (for example ranitidine/cimetidine)	25.73%	115
Prokinetic drug before anesthesia (for example erythromycin or metoclopramide)	23.94%	107
Nombre total de participants: 447		

Q20 You take charge of a 78 year old patient for acute abdominal occlusive syndrome with vomiting (discovery of a colonic neoplasia). How do you manage this case?

Réponses obtenues : 447 Question(s) ignorée(s) : 47

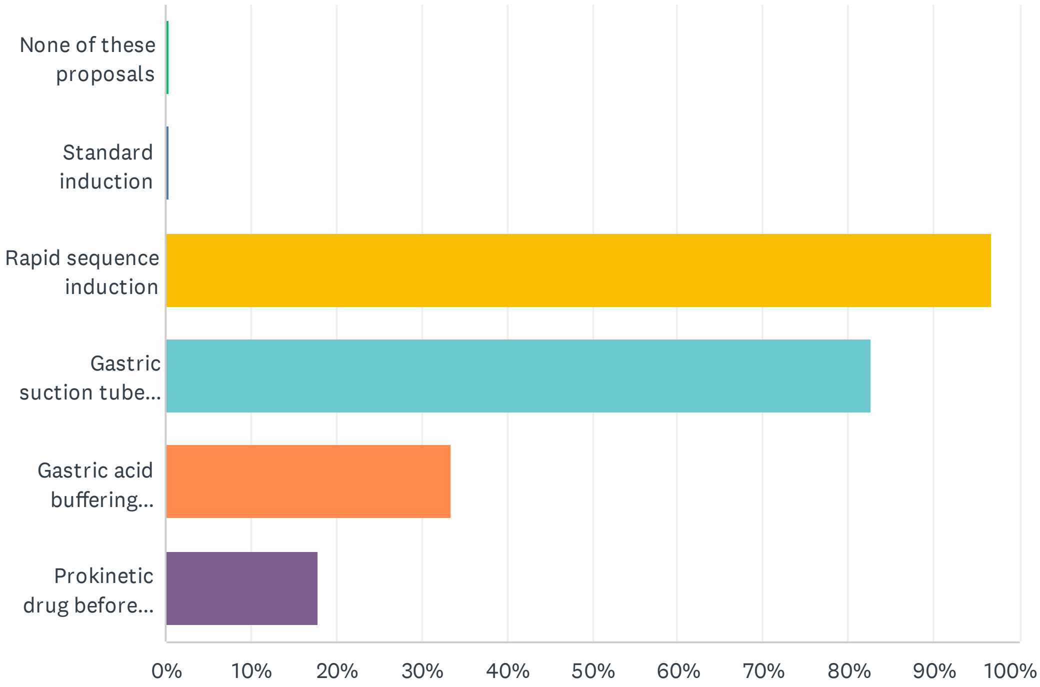

CHOIX DE RÉPONSES	RÉPONSES	
None of these proposals	0.45%	2
Standard induction	0.45%	2
Rapid sequence induction	96.87%	433
Gastric suction tube before anesthesia	82.77%	370
Gastric acid buffering before anesthesia (for example ranitidine/cimetidine)	33.56%	150
Prokinetic drug before anesthesia (for example erythromycin or metoclopramide)	17.90%	80
Nombre total de participants: 447		

Q21 You take charge of a 24 year old patient for a planned caesarean section (placenta percreta) under general anaesthesia. How do you manage this case?

Réponses obtenues : 447 Question(s) ignorée(s) : 47

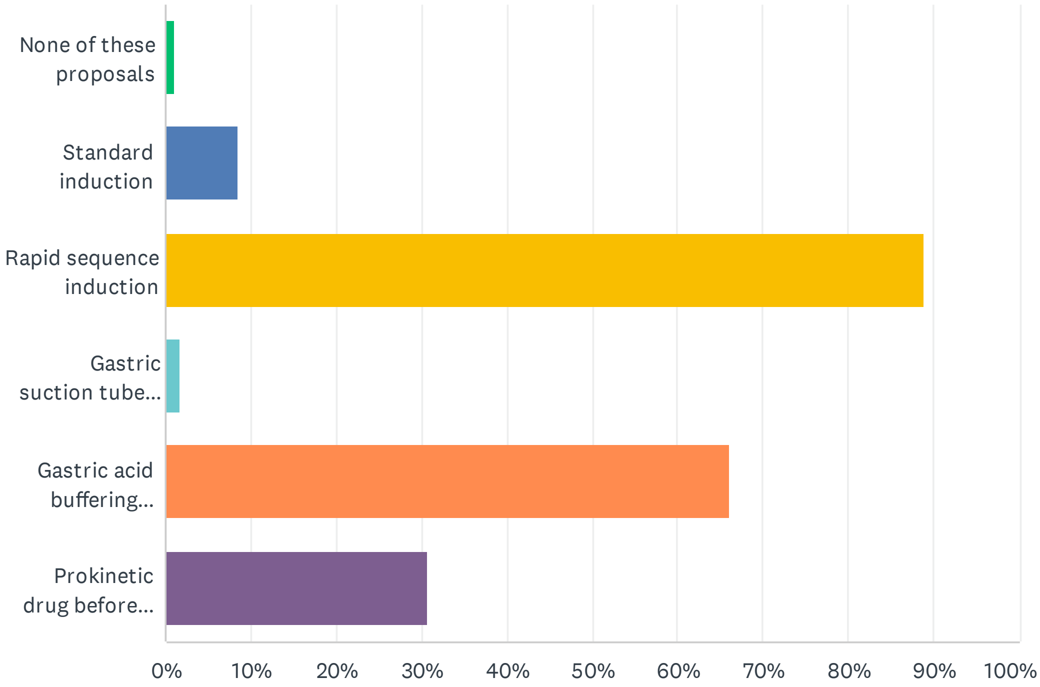

CHOIX DE RÉPONSES	RÉPONSES	
None of these proposals	1.12%	5
Standard induction	8.50%	38
Rapid sequence induction	88.81%	397
Gastric suction tube before anesthesia	1.79%	8
Gastric acid buffering before anesthesia (for example ranitidine/cimetidine)	66.00%	295
Prokinetic drug before anesthesia (for example erythromycin or metoclopramide)	30.65%	137
Nombre total de participants: 447		

Q22 You take charge of a 56 year old patient for thyroidectomy. He has daily untreated and symptomatic gastroesophageal reflux. How do you manage this case?

Réponses obtenues : 447 Question(s) ignorée(s) : 47

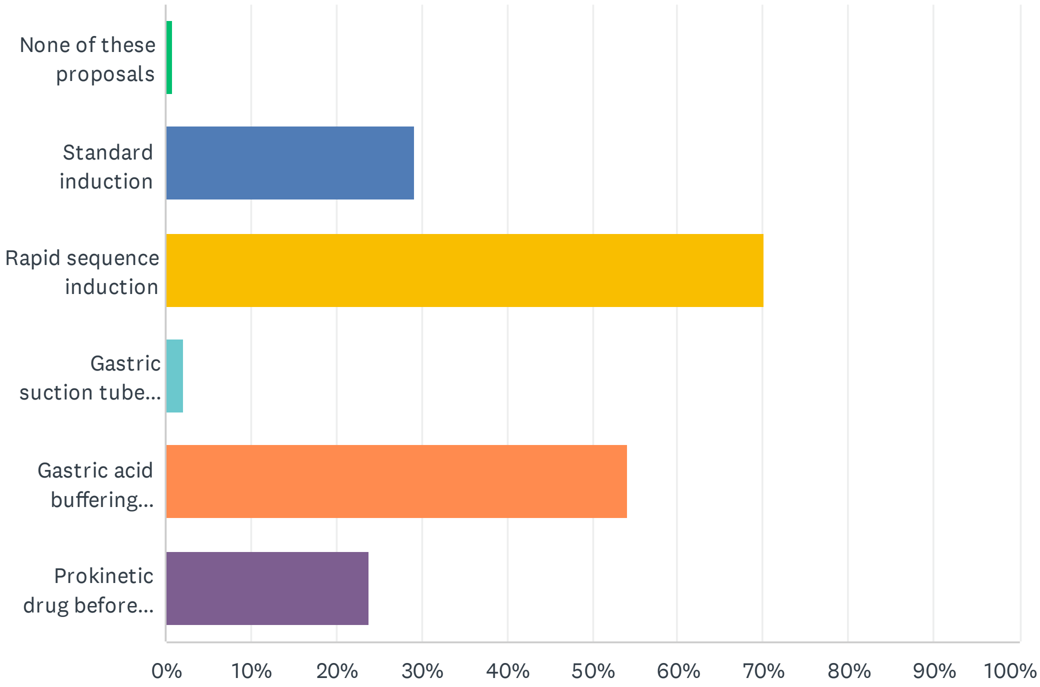

CHOIX DE RÉPONSES	RÉPONSES	
None of these proposals	0.89%	4
Standard induction	29.31%	131
Rapid sequence induction	70.25%	314
Gastric suction tube before anesthesia	2.24%	10
Gastric acid buffering before anesthesia (for example ranitidine/cimetidine)	54.14%	242
Prokinetic drug before anesthesia (for example erythromycin or metoclopramide)	23.94%	107
Nombre total de participants: 447		

Q23 You take charge of a patient aged 56 for thyroidectomy. He has a gastro-oesophageal reflux which is asymptomatic under proton pump inhibitors. How do you manage this case?

Réponses obtenues : 447   Question(s) ignorée(s) : 47

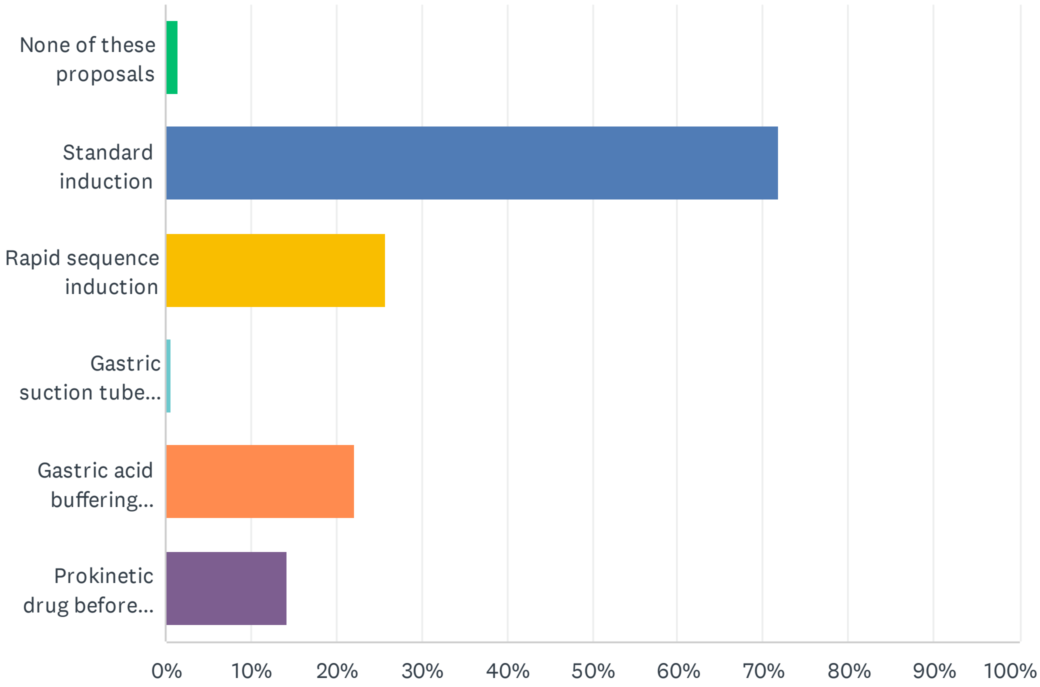

CHOIX DE RÉPONSES	RÉPONSES	
None of these proposals	1.57%	7
Standard induction	71.81%	321
Rapid sequence induction	25.73%	115
Gastric suction tube before anesthesia	0.67%	3
Gastric acid buffering before anesthesia (for example ranitidine/cimetidine)	22.15%	99
Prokinetic drug before anesthesia (for example erythromycin or metoclopramide)	14.32%	64
Nombre total de participants: 447		

Q24 You take charge of a 31 year old patient for a bariatric surgery (gastric bypass). His BMI is 48 kg/m2 with no known gastro-oesophageal reflux. How do you manage this case?

Réponses obtenues : 447   Question(s) ignorée(s) : 47

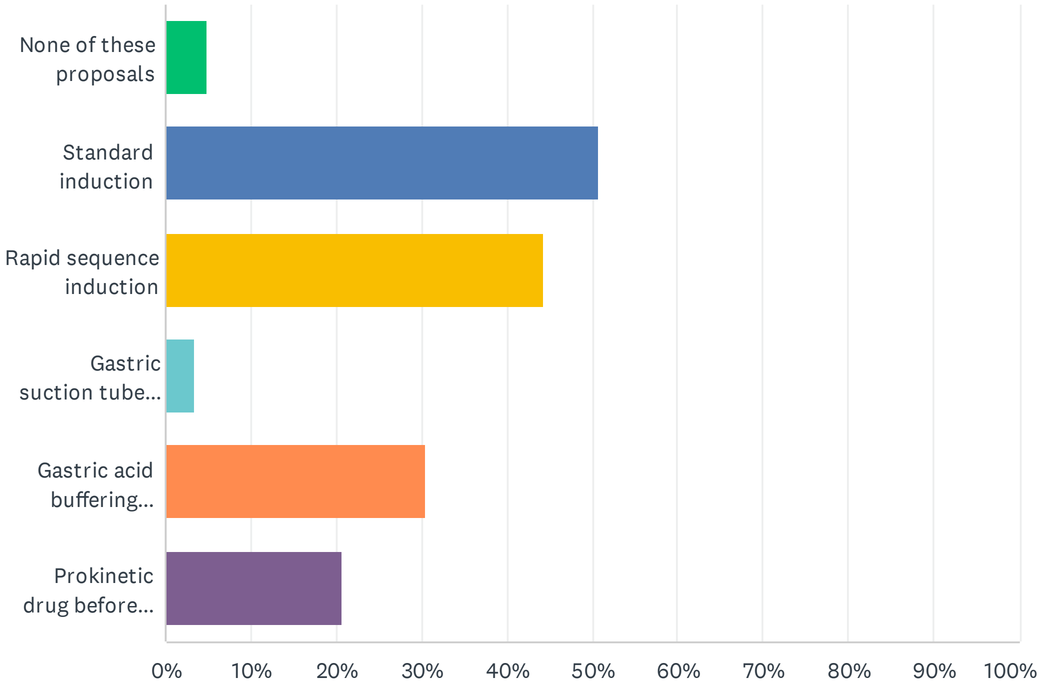

CHOIX DE RÉPONSES	RÉPONSES	
None of these proposals	4.92%	22
Standard induction	50.78%	227
Rapid sequence induction	44.30%	198
Gastric suction tube before anesthesia	3.36%	15
Gastric acid buffering before anesthesia (for example ranitidine/cimetidine)	30.43%	136
Prokinetic drug before anesthesia (for example erythromycin or metoclopramide)	20.58%	92
Nombre total de participants: 447		

Q25 You take charge of a 62 year old patient for a cerebral aneurysmal arterioembolization in a context of a subarachnoid haemorrhage. She has been fasting since the day before and has headaches (pain VAS 5/10) without nausea. How do you manage this case?

Réponses obtenues : 447   Question(s) ignorée(s) : 47

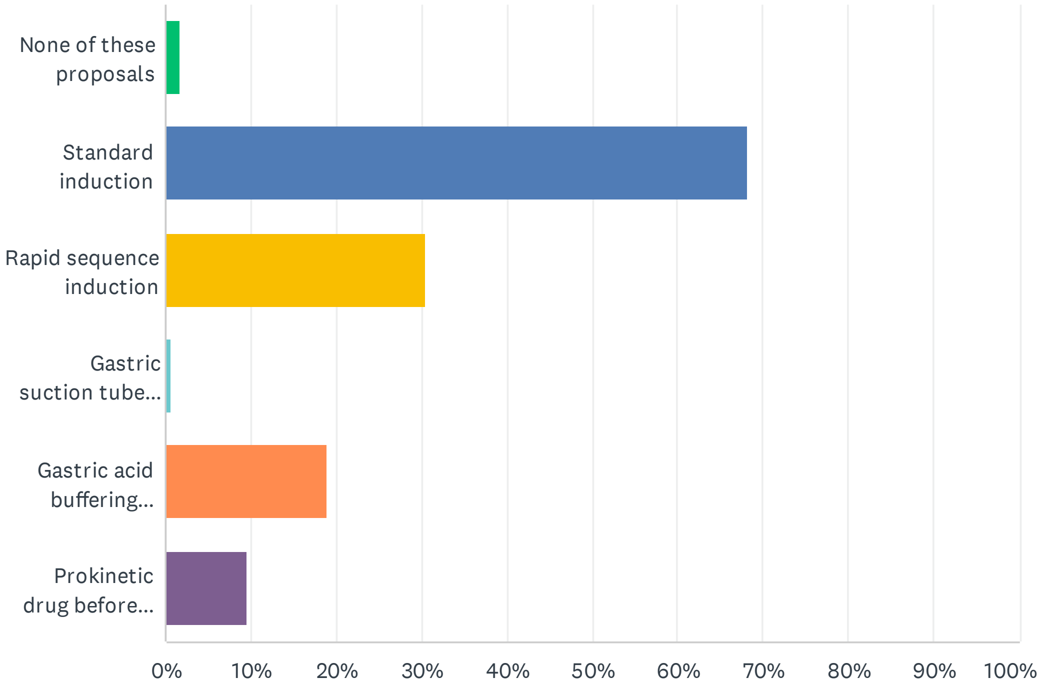

CHOIX DE RÉPONSES	RÉPONSES	
None of these proposals	1.79%	8
Standard induction	68.23%	305
Rapid sequence induction	30.43%	136
Gastric suction tube before anesthesia	0.67%	3
Gastric acid buffering before anesthesia (for example ranitidine/cimetidine)	19.02%	85
Prokinetic drug before anesthesia (for example erythromycin or metoclopramide)	9.62%	43
Nombre total de participants: 447		

Q26 You take charge of a 44 year old patient at 5:00pm, for osteosynthesis of a tibial fracture (time of last meal 7:00am, time of trauma 11:30am). How do you manage this case?

Réponses obtenues : 447 Question(s) ignorée(s) : 47

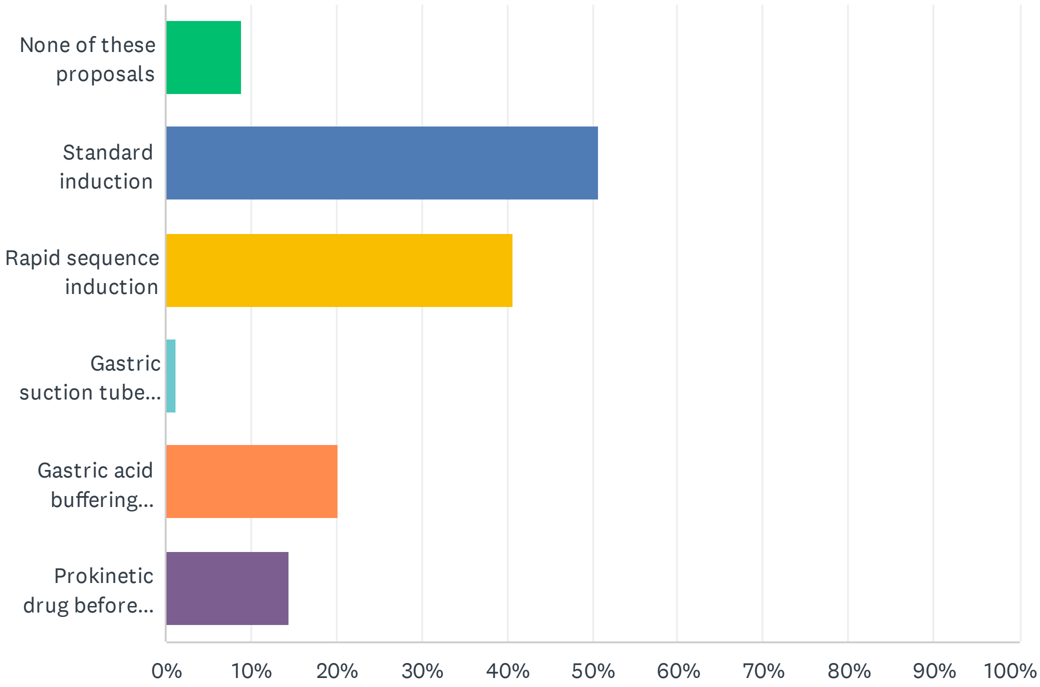

CHOIX DE RÉPONSES	RÉPONSES	
None of these proposals	8.95%	40
Standard induction	50.78%	227
Rapid sequence induction	40.72%	182
Gastric suction tube before anesthesia	1.34%	6
Gastric acid buffering before anesthesia (for example ranitidine/cimetidine)	20.36%	91
Prokinetic drug before anesthesia (for example erythromycin or metoclopramide)	14.54%	65
Nombre total de participants: 447		

Q27 You take charge of a 78 year old patient for scheduled prostate resection. he has an unbalanced diabetes mellitus with no evident sign of gastroparesis. How do you manage this case?

Réponses obtenues : 446 Question(s) ignorée(s) : 48

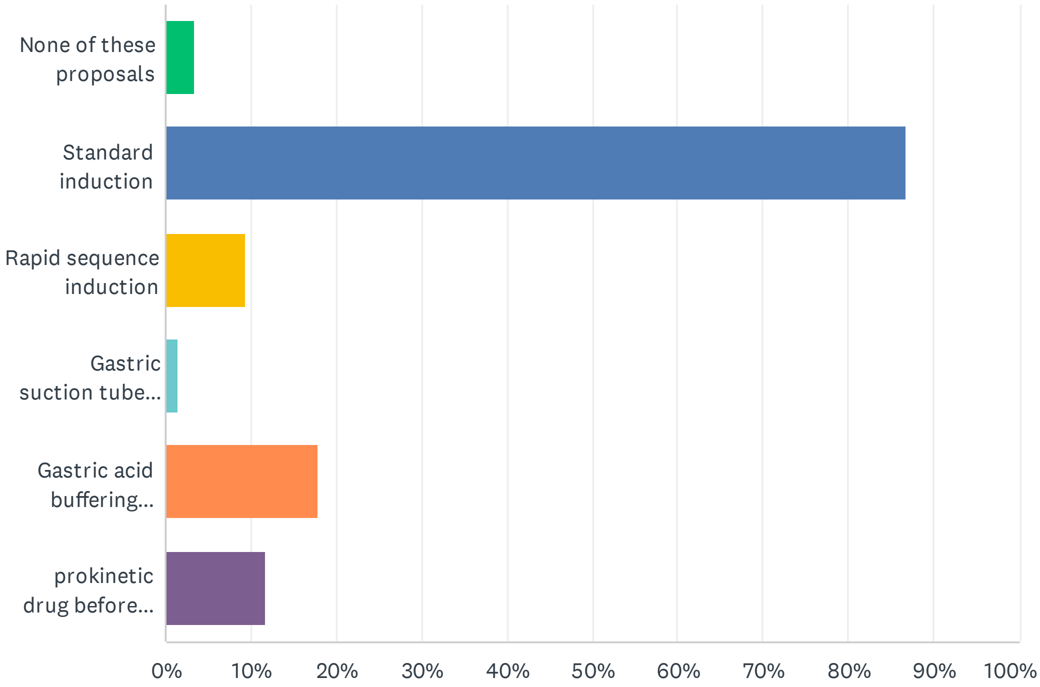

CHOIX DE RÉPONSES	RÉPONSES	
None of these proposals	3.36%	15
Standard induction	86.77%	387
Rapid sequence induction	9.42%	42
Gastric suction tube before anesthesia	1.57%	7
Gastric acid buffering before anesthesia (for example ranitidine/cimetidine)	17.94%	80
prokinetic drug before anesthesia (for example erythromycin or metoclopramide)	11.66%	52
Nombre total de participants: 446		

Q28 Have you ever been directly confronted in your practice with a grade III anaphylactic reaction (shock, bronchospasm) to succinylcholine or rocuronium?

Réponses obtenues : 442 Question(s) ignorée(s) : 52

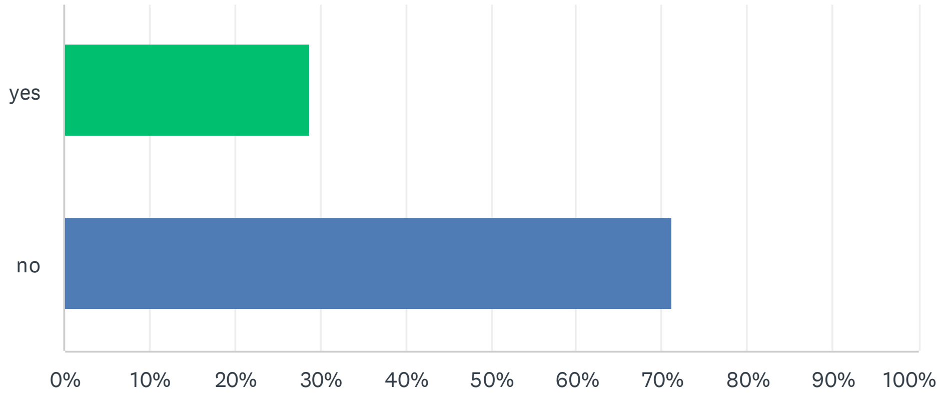

CHOIX DE RÉPONSES	RÉPONSES	
yes	28.73%	127
no	71.27%	315
TOTAL		442

Q29 Have you ever been directly confronted in your practice with a grade IV anaphylactic reaction (cardiac arrest) to succinylcholine or rocuronium?

Réponses obtenues : 443   Question(s) ignorée(s) : 51

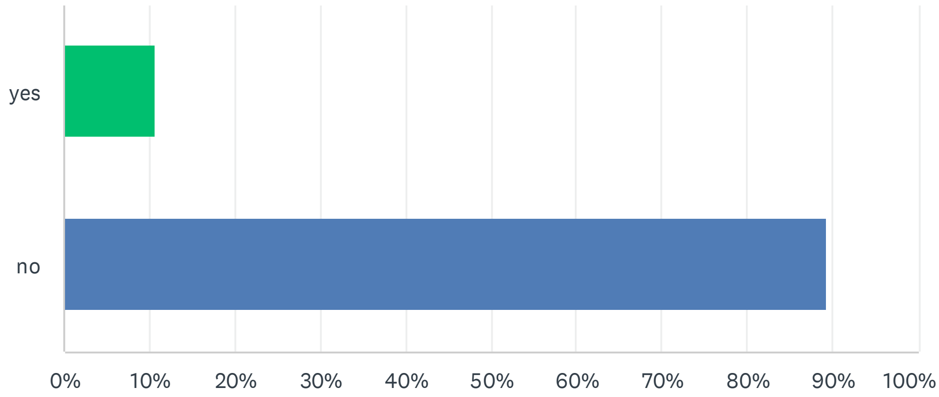

CHOIX DE RÉPONSES	RÉPONSES	
yes	10.61%	47
no	89.39%	396
TOTAL		443

Q30 If yes to questions 28 and/or 29, did the patient die or present major sequels?

Réponses obtenues : 208   Question(s) ignorée(s) : 286

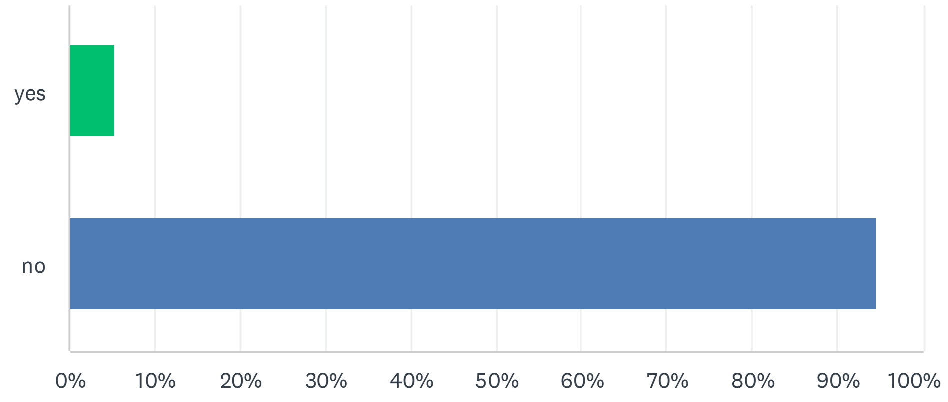

CHOIX DE RÉPONSES	RÉPONSES	
yes	5.29%	11
no	94.71%	197
TOTAL		208

Q31 Have you ever been involved in a legal litigation following an anaphylactic shock situation?

Réponses obtenues : 436   Question(s) ignorée(s) : 58

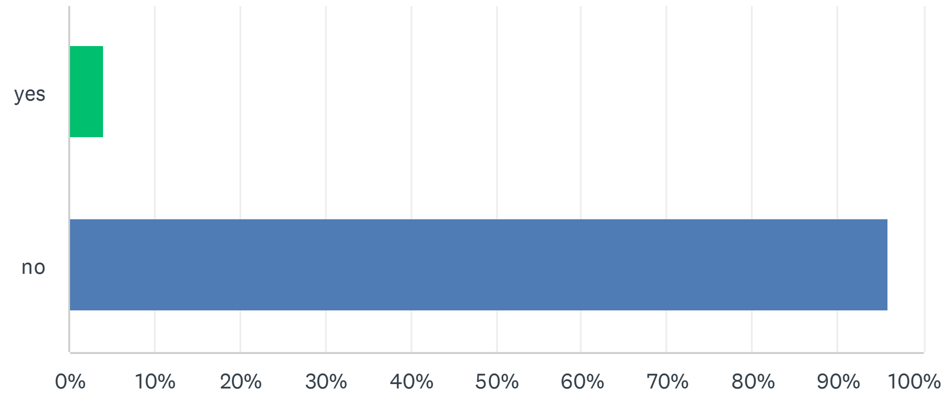

CHOIX DE RÉPONSES	RÉPONSES	
yes	4.13%	18
no	95.87%	418
TOTAL		436

Q32 Have you ever been directly confronted in your practice with an aspiration pneumonia/pneumonitis during anesthesia induction?

Réponses obtenues : 442   Question(s) ignorée(s) : 52

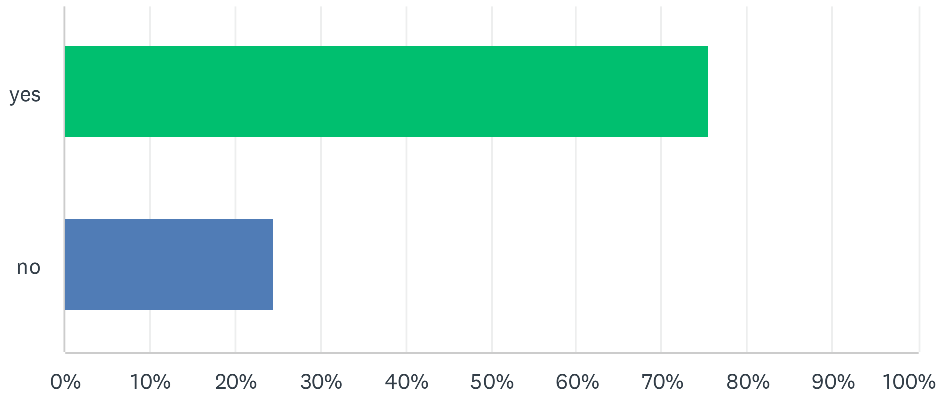

CHOIX DE RÉPONSES	RÉPONSES	
yes	75.57%	334
no	24.43%	108
TOTAL		442

Q33 If yes to the previous question, did a patient die or present major sequels?

Réponses obtenues : 372 Question(s) ignorée(s) : 122

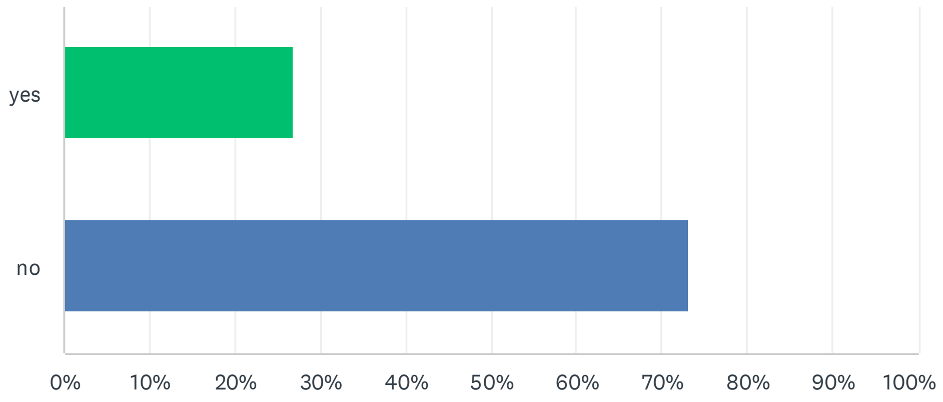

CHOIX DE RÉPONSES	RÉPONSES	
yes	26.88%	100
no	73.12%	272
TOTAL		372

Q34 Have you ever been involved in a legal litigation following an aspiration pneumonia/pneumonitis related to the anesthesia induction?

Réponses obtenues : 440 Question(s) ignorée(s) : 54

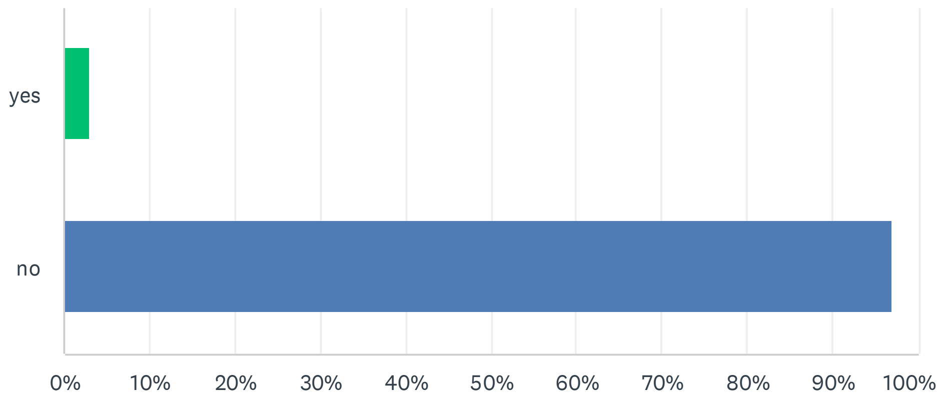

CHOIX DE RÉPONSES	RÉPONSES	
yes	2.95%	13
no	97.05%	427
TOTAL		440
