# Supplementary material for: Practices of Rapid Sequence Induction for Prevention of Aspiration—An International Declarative Survey
Source: J Clin Med. 2025 Mar 22;14(7):2177. doi: 10.3390/jcm14072177 (PMC11989417; doi:10.3390/jcm14072177)
Supplement: Supplementary file 1 [file jcm-14-02177-s001.zip › jcm-3379469-Supplementary File S2.pdf]

Supplementary Table S1 : Responses from different countries (only countries with at least 10 respondents were included)

Country	Number of respondents (% of the total/absolute number)
Austria	4.7% (22)
Belgium	5.2% (24)
Estonia	4.1% (19)
France	5.6% (26)
Germany	10.2% (47)
Greece	2.8% (13)
Italy	3.4% (16)
Netherlands	3.9% (18)
Portugal	5.6% (26)
Spain	5.8% (27)
Sweden	2.8% (13)
Switzerland	8.2 % (38)
India	2.6 % (12)
Turkey	3.2% (15)
UK	2.3% (11)
**Total**	**327 respondents (66% of the whole population)**

Supplementary Table S2: Rapid sequence induction definitions according to country respondents

Country	Short acting hypnotic+ Short acting NMB (Succinylcholine/ Rocuronium)	Short acting hypnotic+ Short acting NMB+ opioid	Short acting hypnotic alone	Short acting hypnotic+ opioid
Austria	**40.9% (9)**	**95.4% (21)**	**0**	**0**
Belgium	**33.3 % (8)**	**87.5% (21)**	**0**	**0**
Estonia	**31.5% (6)**	**73.6% (14)**	**0**	**0**
France	**76.9% (20)**	**57.6% (15)**	**0**	**0**
Germany	**21.2%(10)**	**87.2% (41)**	**0**	**0**
Greece	**46.1%(6)**	**76.9% (10)**	**0**	**0**
Italy	**43.7% (7)**	**68.7% (11)**	**0**	**0**
Netherlands	**66.6% (12)**	**50% (9)**	**0**	**0**
Portugal	**38.4% (10)**	**80.7% (21)**	**0**	**0**
Spain	**66.6% (18)**	**59.2% (16)**	**0**	**3.7% (1)**
Sweden	**23% (3)**	**100% (13)**	**0**	**0**
Switzerland	**39.4% (15)**	**73.6% (28)**	**5.2% (2)**	**0**
India	**66.6% (8)**	**33.3% (4)**	**0**	**0**
Turkey	**80% (12)**	**20% (3)**	**6.6% (1)**	**0**
UK	**45.4% (5)**	**54.4% (6)**	**0**	**0**

Supplementary Table S3: Preoxygenation methods before rapid sequence induction

Country	High flow oxygen (> 10L/min), no PEEP, no pressure support	Non-invasive ventilation with pressure support, no PEEP (< 3 cmH2O)	Non-invasive ventilation with pressure support AND PEEP (>= 3 cmH2O	No preoxygenation	High flow nasal canula	Other (please specify)
Austria	**100 % (22)**	**13.6 % (3)**	**22.7% (5)**	**0**	**45% (10)**	**4.5 % (1)**
Belgium	**100% (24)**	**4.1% (1)**	**8.2 % (2)**	**0**	**16.4% (4)**	**12.5 % (3)**
Estonia	**89.4% (17)**	**10.5% (2)**	**26.3% (5)**	**0**	**21% (4)**	**10.5% (2)**
France	**84.6% (22)**	**30.7 % (8)**	**34. 6 (9)**	**0**	**11.5% (3)**	**0**
Germany	**80.8% (38)**	**6.3% (3)**	**25.5% (12)**	**0**	**19.1% (9)**	**8.5% (4)**
Greece	**84.6% (11)**	**7.6% (1)**	**23% (3)**	**0**	**15.3% (2)**	**0**
Italy	**81.2% (13)**	**6.2% (1)**	**18.7% (3)**	**0**	**0**	**0**
Netherlands	**94.4% (17)**	**11.1% (2)**	**5.5% (1)**	**0**	**0**	**0**
Portugal	**69.2% (18)**	**3.8% (1)**	**19.2% (5)**	**0**	**23% (6)**	**0**
Spain	**85.1% (23)**	**3.7% (1)**	**14.8% (4)**	**0**	**14.8% (4)**	**7.4% (2)**
Sweden	**100% (13)**	**0**	**30.7% (4)**	**0**	**15.3% (2)**	**0**
Switzerland	**81.5% (31)**	**15.7% (6)**	**23.6 % (9)**	**0**	**28.9% (11)**	**5.2% (2)**
India	**91.6% (11)**	**8.3 (1)**	**0**	**0**	**16.6% (2)**	**0**
Turkey	**93.3% (14)**	**6.6% (1)**	**0**	**0**	**26.6% (4)**	**0**
UK	**54.5% (6)**	**0**	**9% (1)**	**0**	**9% (1)**	**0**

Supplementary Table S4: Rapid sequence induction side effects and related legal litigations

Country	RSI side effects			Aspiration		Legal litigations	
	Anaphylaxis grade 3	Anaphylaxis grade 4 (cardiac arrest)	Major issues due to anaphylaxis (including mortality)	Occurrence	Major issues due to aspiration (including mortality)	Related to anaphylaxis	Related to aspiration
Austria	**27.2% (6)**	**13.6% (3)**	**0**	**72.7% (16)**	**27.2% (6)**	**4.5% (1)**	**4.5% (1)**
Belgium	**45.8% (11)**	**25% (6)**	**8.3% (2)**	**83.3% (20)**	**20.8% (5)**	**0**	**0**

Estonia	**15.7% (3)**	**5.2% (1)**	**0**	**78.9% (15)**	**10.5% (2)**	**10.5% (2)**	**10.5% (2)**
France	**57.6% (15)**	**34.6 % (9)**	**11.5% (3)**	**69.2% (18)**	**11.5% (3)**	**11.5% (3)**	**0**
Germany	**19.1 % (9)**	**10.6% (5)**	**0**	**82.9% (39)**	**31.9% (15)**	**4.2% (2)**	**2.1% (1)**
Greece	**30.7% (4)**	**15.3% (2)**	**7.6% (1)**	**61.5 % (8)**	**15.3% (2)**	**0**	**0**
Italy	**12.5 (2)**	**0**	**0**	**56.2% (9)**	**6.2% (1)**	**0**	**0**
Netherlands	**16.6% (3)**	**11.1% (2)**	**5.5% (1)**	**83.3% (15)**	**27.7% (5)**	**0**	**0**
Portugal	**15.3% (4)**	**7.6% (2)**	**0**	**69.2% (18)**	**11.5% (3)**	**0**	**0**
Spain	**3.7% (5)**	**0**	**0**	**66.6% (18)**	**14.8% (4)**	**0**	**3.7% (1)**
Sweden	**23% (3)**	**0**	**0**	**61.5% (8)**	**23% (3)**	**0**	**0**
Switzerland	**42.1% (16)**	**13.1% (5)**	**0**	**92.1% (35)**	**36.8% (14)**	**2.6% (1)**	**2.6% (1)**
India	**8.5% (1)**	**0**	**0**	**25% (3)**	**8.5% (1)**	**0**	**0**
Turkey	**13.3% (2)**	**0**	**0**	**40% (6)**	**13.3% (2)**	**13.3% (2)**	**6.6% (1)**
UK	**45.4% (5)**	**9% (1)**	**9 % (1)**	**45.4 % (5)**	**18.1% (2)**	**0**	**0**
